# Mechanisms and Molecular Targets of the Tao-Hong-Si-Wu-Tang Formula for Treatment of Osteonecrosis of Femoral Head: A Network Pharmacology Study

**DOI:** 10.1155/2020/7130105

**Published:** 2020-09-09

**Authors:** Fanyu Fu, Zeqing Huang, Hengli Ye, Biao Tan, Rongtian Wang, Weiheng Chen

**Affiliations:** ^1^Guizhou University of Traditional Chinese Medicine, No. 50, Shidonglu, Nanming District, Guizhou, Guiyang 550002, China; ^2^Department of Orthopaedics, Wangjing Hospital, China Academy of Chinese Medical Sciences, No. 6 Zhonghuannanlu, Chaoyang District, Beijing 100102, China; ^3^Department of Orthopaedics, The Third Affiliated Hospital of Beijing University of Chinese Medicine, No. 51 Xiaoguan Street, Chaoyang District, Beijing 100029, China

## Abstract

The Tao-Hong-Si-Wu-Tang (THSWT) formula, a classic prescription of traditional Chinese medicine, has long been used for the treatment of osteonecrosis of femoral head (ONFH). However, its mechanisms of action and molecular targets are not comprehensively clear. In the present study, the Traditional Chinese Medicine System Pharmacology (TCMSP) database was employed to retrieve the active compounds of each herb included in the THSWT formula. After identifying the drug targets of active compounds and disease targets of ONFH, intersection analysis was conducted to screen out the shared targets. The protein-protein network of the shared targets was built for further topological analysis. Gene ontology and Kyoto Encyclopedia of Genes and Genomes pathway analysis were then carried out. A gene pathway network was constructed to screen the core target genes. We identified 61 active compounds, 155 drug targets, and 5443 disease targets. However, intersection analysis only screened out 37 shared targets. Kaempferol, luteolin, and baicalein regulated the greatest number of targets associated with ONFH. The THSWT formula may regulate osteocyte function through specific biological processes, including responses to toxic substances and oxidative stress. The regulated pathways included the relaxin, focal adhesion, nuclear factor-*κ*B, toll-like receptor, and AGE/RAGE signaling pathways. *RELA*, *VEGFA*, and *STAT1* were the important target genes in the gene network associated with the THSWT formula for the treatment of ONFH. Therefore, the present study suggested that the THSWT formula has an action mechanism involving multiple compounds and network targets for the treatment of ONFH.

## 1. Introduction

Osteonecrosis of femoral head (ONFH) represents a disruption of the blood supply to the femoral head due to trauma, corticosteroids, alcohol, and other ill-defined etiologies [1]. ONFH mainly affects individuals of working age [2]. It is estimated that there are more than 8 million patients with nontraumatic ONFH in China [3]. According to natural history studies, approximately half of all affected hips at pre-collapse stages (Association Research Circulation Osseous stage I or II) [4] would progress to irreversible collapse of the femoral head if left untreated [5]. Femoral head collapse can then progress to severe premature osteoarthritis of the hip, which is a common cause of lifelong disability and total hip arthroplasty in this active population [6].

To date, there are no optimal treatments for ONFH [7]. Total hip arthroplasty is not the first-choice treatment option since revision procedures and implant longevity remain tricky problems [8]. Other treatment modalities, commonly known as hip-preserving treatments, have demonstrated both favorable and poor outcomes [9, 10]. According to a meta-analysis published in 2019, no marketed drugs are recommended for the treatment of ONFH [11].

During the past decade, Chinese experts have developed four versions of clinical guidelines, and traditional Chinese medicine (TCM) is consistently recommended as one of the main nonoperative treatments [12–15]. TCM holds a relatively unique point of view when treating ONFH, and blood stasis (Yu-Xue) is considered the pathological basis of ONFH [16]. According to the TCM theory, once the meridian branches (Jing-Luo) passing through the femoral head are blocked, the femoral head loses nutrition from qi and blood. Based on the blood stasis theory, the primary management strategy of TCM is to activate blood circulation (Huo-Xue-Fa) [15]. The Tao-Hong-Si-Wu-Tang (THSWT) formula is composed of Tao-Ren (*Persicae Semen*), Hong-Hua (*Carthami Flos*), Dang-Gui (*Angelicae Sinensis Radix*), Chuang-Xiong (*Chuanxiong Rhizoma*), Shu-Di-Huang (*Rehmanniae Radix Praeparata*), and Bai-Shao (*Paeoniae Radix Alba*), of which the main TCM function is to activate blood circulation. The THSWT formula is frequently administered in patients with ONFH in China. Data from animal testing suggest that the THSWT formula may help ameliorate the progression of steroid-induced avascular necrosis [17]. However, the active compounds and potential targets, as well as action pathways, remain poorly understood.

A general solution related to network pharmacology has been proposed recently, which has become a hot topic to investigate multiple molecular mechanisms of multiple-target compounds affecting biological networks for herbal medicines. Therefore, we employed network pharmacology to probe the pharmacological mechanisms of the THSWT formula against ONFH in this study.

## 2. Materials and Methods

### 2.1. Screening of Active Compounds

The THSWT formula consists of six Chinese herbs, including Tao-Ren (*Persicae Semen*), Hong-Hua (*Carthami Flos*), Dang-Gui (*Angelicae Sinensis Radix*), Chuang-Xiong (*Chuanxiong Rhizoma*), Shu-Di-Huang (*Rehmanniae Radix Praeparata*), and Bai-Shao (*Paeoniae Radix Alba*). The chemical compounds of these six herbs were identified using the Chinese Medicine System Pharmacology Database and Analysis Platform (TCMSP, http://tcmspw.com/tcmsp.php) [18]. TCMSP provides important data on the absorption-, distribution-, metabolism-, and excretion-related properties of Chinese herbs, including the oral bioavailability (OB), half-life, and drug-likeness (DL). In the present study, chemical compounds with OBs ≥30% and DLs ≥0.18 were identified as active compounds. Eventually, 61 active compounds were screened out in total after removing duplications.

### 2.2. Identification of Drug Targets

The DrugBank (http://www.drugbank.ca) [19] was employed to investigate potential targets of the 61 selected compounds. The DrugBank is a database containing approved drugs as well as experimental drugs. Finally, 587 drug targets were identified, including 102 in *Persicae Semen*, 257 in *Carthami Flos*, 55 in *Angelicae Sinensis Radix*, 30 in *Rehmanniae Radix Praeparata*, 104 in *Paeoniae Radix Alba*, and 39 in *Chuanxiong Rhizoma*. A total of 155 drug targets were collected after removing duplications. Protein sequences of these drug targets were normalized to official gene symbols using the UniProt database (https://www.uniprot.org/) [20].

### 2.3. Identification of Disease Targets

The differentially expressed genes associated with ONFH were downloaded from the GEO database (https://www.ncbi.nlm.nih.gov/geo/, Series: GSE74089, Samples: GSM1909502, GSM1909503, GSM1909504, GSM1909505, GSM1909506, GSM1909507, GSM1909508, GSM1909509) [21]. These original data were converted into a gene matrix using the Perl tool [22]. The collated data were analyzed using the Limma plugin of R software. Genes with a *P*-value <0.05 and |log_2 _(fold change)| >1 were identified as disease targets of ONFH.

### 2.4. Protein-Protein Interaction Network Construction

The Venny 2.1 online tool (http://bioinfogp.cnb.csic.es/tools/venny/index.html) was used to draw a Venn diagram of drug targets and disease targets to obtain shared targets of the THSWT formula and ONFH. The shared target genes were then inputted into the String database (http://string-db.org), with species limited to *Homo sapiens* and a confidence score >0.4, to construct the protein-protein interaction (PPI) network. The PPI network of drug targets and disease targets was visualized using Cytoscape software. Maximum Clique Centrality (MCC) is a network topology algorithm of the Cytohubba plugin, which helps identify core targets in the network. In the present study, the top 10 targets with the highest MCC scores were considered the core targets of the THSWT formula against ONFH.

### 2.5. Network Construction Method

An active compound-shared target network was constructed and visualized using Cytoscape 3.7.2 software. The core compounds and core targets in this network were automatically identified. Each node in the network represented an active compound or shared target. The edge between two nodes implied that a particular compound might act on the target connected with it. The topological parameters of each node, including the degree, betweenness, and closeness, were calculated and used as screening criteria for the crucial nodes. Overall, nodes with greater parameter values were recognized as crucial nodes of the THSWT formula against ONFH. In the present study, a key compound was required to fulfill the criterion that these three parameters exceeded the median of the selected compounds.

### 2.6. Bioinformatics Analysis

Gene ontology (GO) biological process (BP) enrichment analysis and Kyoto Encyclopedia of Genes (KEGG) pathway enrichment analysis were conducted using the David 6.8 database (https://david.ncifcrf.gov/). During these procedures, P.adjust <0.05 suggested statistical significance in the enrichment degree. The top 20 GO and top 20 KEGG results with the lowest P.adjust values were displayed in the form of bubble charts using R-studio software. The genes with significantly regulated pathways were selected for gene pathway network analysis to screen out the key target genes of the THSWT formula in the treatment of ONFH.

## 3. Results

### 3.1. Active Compounds and Shared Targets

**Figure 1:**
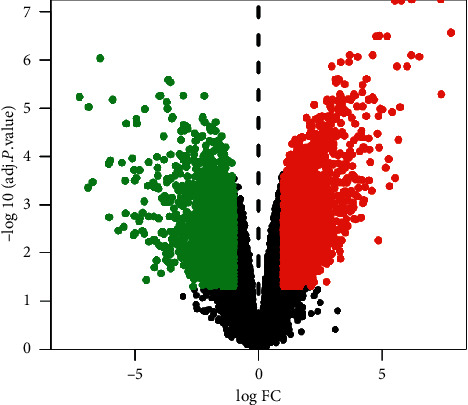
Volcano map of differently expressed genes. Green dots represent the downregulated genes, and red dots represent the upregulated genes.

**Figure 2:**
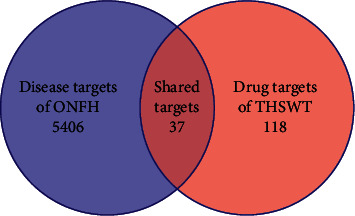
Venn diagram showing the intersection of drug targets of the THSWT formula and disease targets of osteonecrosis of femoral head.

Sixty-one chemical compounds of the THSWT formula (Table 1) were identified as the active compounds. The distribution of differentially expressed genes was displayed using volcanic maps (Figure 1). Data of upregulated genes were shown as red dots, and downregulated genes were shown as green dots. A total of 5443 differentially expressed genes in ONFH were collected from the GEO database, including 3291 upregulated genes and 2152 downregulated genes. Intersection analysis of 155 drug target genes and 5443 disease target genes identified 37 shared targets (Figure 2). These 37 targets were considered potential targets of the THSWT formula for the treatment of ONFH.

### 3.2. PPI Network Analysis

**Figure 3:**
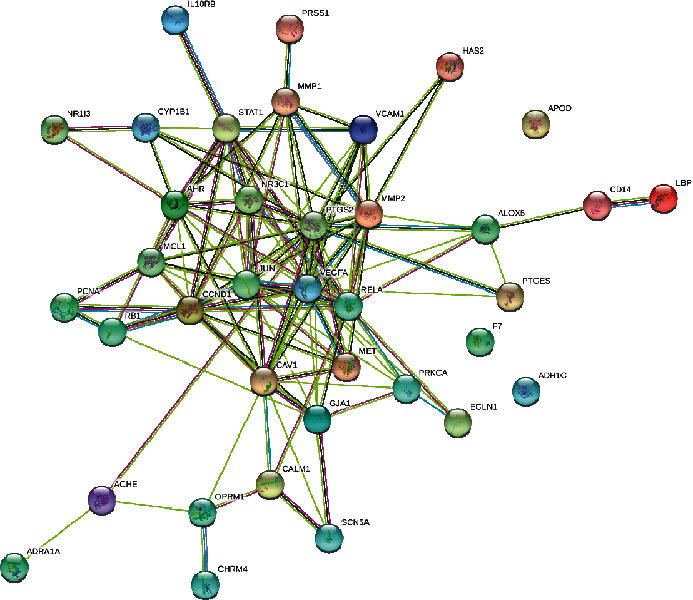
Protein-protein interaction network of the 37 shared targets.

**Figure 4:**
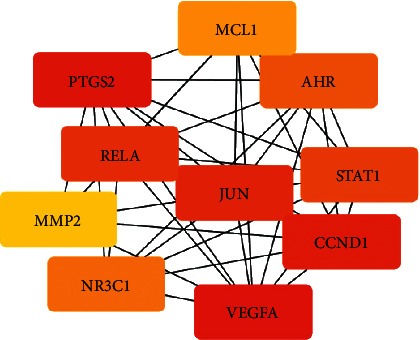
Protein-protein interaction network of the top 10 shared targets with highest MCC scores. The shade of color is proportional to the topological importance, with red targets indicating more important positions.

The PPI network (Figure 3) contained 37 nodes, which corresponded to 37 shared targets, and 120 edges that represented the target-target interactions. The top 10 target genes with the highest MCC scores were *VEGFA*, *PTGS2*, *CCND1*, *JUN*, *RELA*, *STAT1*, *AHR*, *NR3C1*, *MCL1*, and MMP2, which were considered the core targets (Figure 4).

### 3.3. Compound-Shared Target Network Analysis

**Figure 5:**
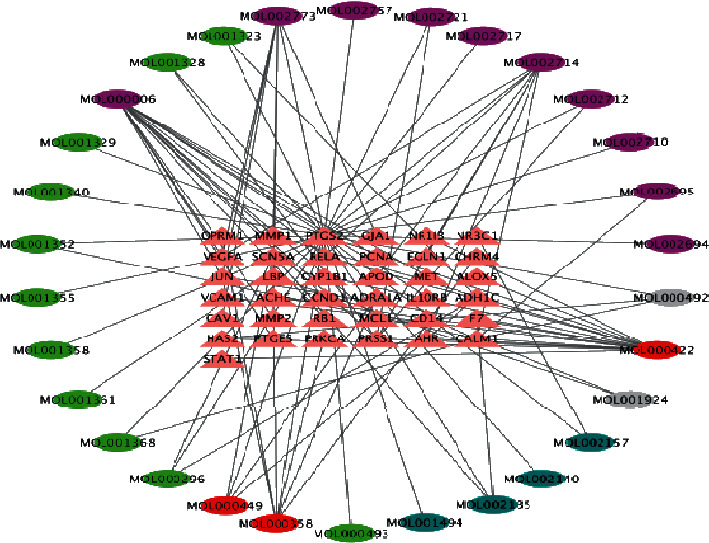
The compound-shared targets network. The ovals represent the chemical compounds, the colors represent the herb sources of chemical compounds, and the pink triangles represent the 37 shared genes. The gray ovals represent *Paeoniae Radix Alba*, the dark green ovals represent *Chuanxiong Rhizoma*, the purple ovals represent *Carthami Flos*, the light green ovals represent *Persicae Semen*, and the red ovals represent chemical compounds with multiple herb sources.

The compound-shared target network (Figure 5) contained 67 nodes, which corresponded to 30 candidate compounds, 37 shared targets, and 94 edges representing the compound-target interactions (Table 2). Topological calculations revealed nine compounds fulfilling the criteria with all parameter values (degree, betweenness, and closeness) exceeding the median of the 30 selected compounds (Table 3). Overall, kaempferol, luteolin, and baicalein were found to act on the top three greatest numbers of targets (15, 14, and 8 targets, respectively). In addition, the OBs of kaempferol, luteolin, and baicalein were 41.88%, 36.16%, and 33.52%, respectively. Therefore, they were considered the key compounds in the THSWT formula for the treatment of ONFH.

### 3.4. GO and KEGG Pathway Enrichment Analyses

**Figure 6:**
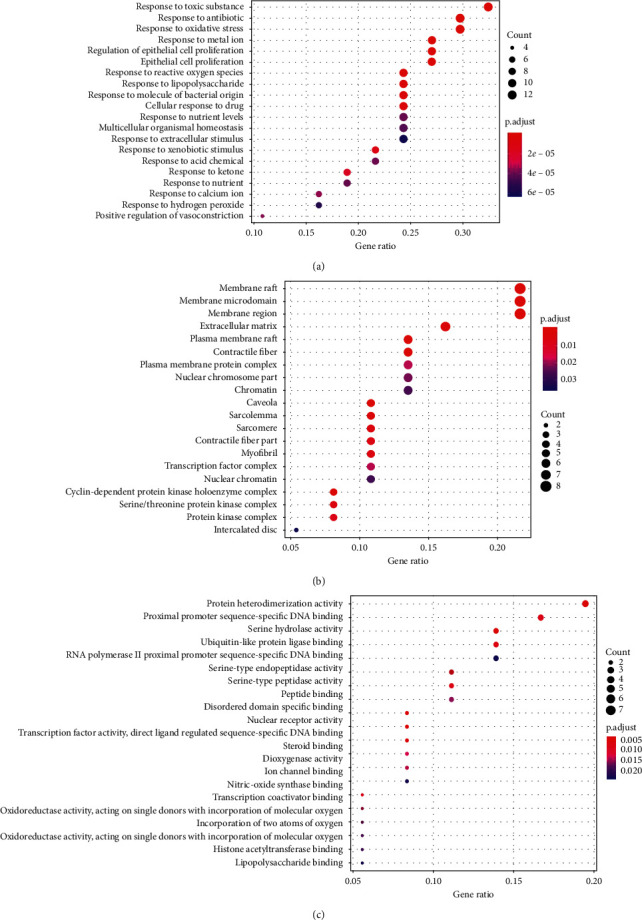
Gene ontology terms of candidate targets of the THSWT formula against ONFH. A, biological process. B, cellular component. C, molecular function.

BP, cellular component (CC), and molecular function (MF) analyses of the 37 target genes revealed 603 GO terms that were significantly enriched, including 540 in BP, 23 in CC, and 40 in MF analyses. The GO terms with the top 20 lowest P.adjust values are shown in Figure 6.

**Figure 7:**
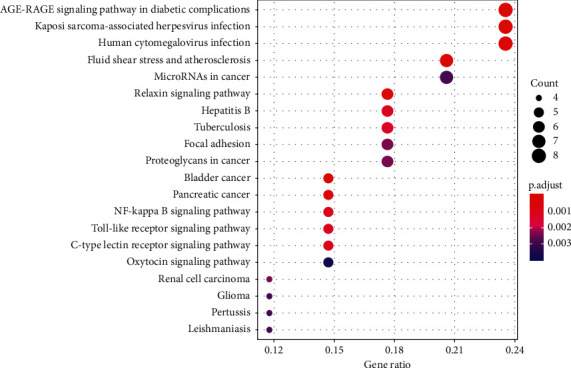
KEGG pathway enrichment of candidate targets of the THSWT formula against ONFH.

KEGG pathway analysis revealed 78 pathways that were significantly enriched. The top 20 terms are shown in Figure 7. The clinically significant pathways in the top 20 included the relaxin, focal adhesion, nuclear factor (NF)-*κ*B, Toll-like receptor (TLR), and AGE/RAGE signaling pathways.

**Figure 8:**
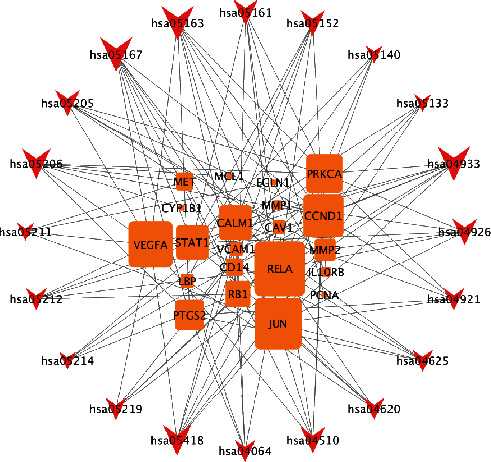
Gene-pathway network of the THSWT formula against ONFH. The orange squares represent target genes and the red V-shapes represent pathways. The importance of targets and pathways is proportional to their shape sizes.

Finally, the gene pathway network was constructed based on the significantly enriched pathways and genes that regulated these pathways, as presented in Figure 8. Topological analysis of 20 pathways and 21 genes was carried out. The squares represented target genes, and the V-shapes represented pathways in the network. The network diagram suggested that *RELA* had the maximum degree (number of connected nodes) and thus was considered the core target. Several other targets also had more significant degrees, such as *JUN*, *VEGFA*, and *CCND1*.

## 4. Discussion

TCM holds a similar view that ischemia of the femoral head is a key pathological change in ONFH. Chinese herbal medications with the TCM function of activating blood (Huo-Xue-Fa) have been consistently recommended by Chinese guidelines as an important nonoperative treatment for ONFH [12–15]. The THSWT formula, as a basic prescription to implement the therapeutic principle of activating blood [23], has demonstrated promising effects in ameliorating the progression of ONFH [24]. However, the biological activity of the THSWT formula remains poorly understood, particularly regarding whether it can increase the blood supply to the femoral head and whether it possesses any bone protective activity. Data from the present study suggest that the THSWT formula contains multiple active compounds that act on a network of different targets by regulating a number of signaling pathways, which contribute to the implementation of the THSWT formula in clinical practice.

An updated meta-analysis concluded that marketed drugs fail to prevent the progression of ONFH [11], but an increasing number of clinical studies on TCM have demonstrated promising outcomes [25, 26]. Essentially, TCM prescribes several natural compounds, most of which are still not approved as marketed productions. However, this can be an important way to discover potential drugs for ONFH. In the present study, kaempferol, luteolin, and baicalein were among the important active compounds of the THSWT formula, since these compounds can act on 15, 14, and 8 different disease targets, respectively. Kaempferol is a common flavonol present in Chinese herbs with therapeutic properties, including antioxidant and anti-inflammatory activities [27]. Recent studies have suggested that kaempferol also has bone protective activity, since animal testing has found that kaempferol antagonizes the apoptotic effect of dexamethasone on osteoblasts [28]. Both isolated luteolin and extracts from luteolin-rich plants exhibit anti-inflammatory activity [29]. Luteolin also helps inhibit the bone resorption induced by mature osteoclasts [30]. A number of studies have demonstrated that baicalein has potent neuroprotective properties [31]. Additionally, baicalein inhibits the bone resorptive activity of mature osteoclasts by inducing apoptosis [32]. We can easily conclude that the natural compounds of the THSWT formula, particularly the three aforementioned compounds, confer bone protective activity and have high OB scores; they are likely to be the core compounds for the treatment of ONFH.

GO enrichment analysis suggested that the THSWT formula regulates a variety of BPs and affects various CCs and MFs. Cellular responses to toxic substances and oxidative stress are important BPs involved in the development of ONFH. Corticosteroids and alcohol are key toxic substances that cause ONFH. Previous studies have confirmed that the rs1045642 single-nucleotide polymorphism of *ABCB1*, an important determinant in the elimination of toxic substances, is closely associated with the occurrence of steroid-induced ONFH [33]. Moreover, oxidative stress plays a role in the activation of coagulation, which is the underlying BP that leads to ischemia of the femoral head [34]. Our data showed that membrane raft and membrane microdomains are among the most significant CCs affected by the THSWT formula. Additionally, the significantly mediated MFs include protein heterodimerization activity and proximal promoter sequence-specific DNA binding.

KEGG enrichment analysis suggested that the THSWT formula may regulate various signaling pathways. The relaxin, focal adhesion, and NF-*κ*B signaling pathways are enriched pathways with important clinical significance. The relaxin signaling pathway is a potent stimulator of osteoclastogenesis from hematopoietic precursors, which regulate the activity of mature osteoclasts [35]. Focal adhesion kinase (FAK) is a nonreceptor protein tyrosine kinase and scaffolding protein that mediates numerous cellular functions, including adhesion, migration, and invasion. FAK inhibitors reduce synovial fibroblast invasion and migration [36]; thus, inhibition of FAK may help ameliorate the bone marrow edema and synovitis observed in the development of ONFH. TLR antagonists can be used for the treatment of inflammatory and autoimmune diseases, which also inhibit the activation of NF-*κ*B. NF-*κ*B, one of the most important transcriptional signaling molecules, participates in downstream inflammatory pathways and the TLR signaling pathway. The essential role of NF-*κ*B in osteoclastogenesis has been demonstrated genetically. NF-*κ*B can transduce signals by recruiting adaptor molecules. In addition, NF-*κ*B can induce the proliferation of monocytes/macrophages, which finally form osteoclasts [37]. Our data also suggested that the biological activity of the THSWT formula is associated with a number of pathways involved in other diseases, including infections, cancers, and diabetes-related complications. Interestingly, the AGE/RAGE signaling pathway is the most enriched pathway based on our data. AGE/RAGE signaling is a well-studied cascade in many different disease states; inhibition of the AGE/RAGE system may be a promising target for therapeutic intervention for vascular complications such as acquired blindness, end-stage renal failure, a variety of neuropathies, and accelerated atherosclerosis [38]. The AGE/RAGE signaling pathway also plays an important physiological role in the regulation of skeletal development, homeostasis, and repair/regeneration [39].

Gene pathway network analysis revealed that *RELA*, *VEGFA*, and *STAT1* were among the core targets of the THSWT formula in the treatment of ONFH. RELA is a member of the NF-*κ*B/Rel family. The transcription factor NF-*κ*B is a critical regulator of immune and inflammatory responses. Mice lacking RelA/p65 in the hematopoietic compartment have been shown to have a deficient osteoclastogenic response to RANKL and are protected from arthritis-induced osteolysis. It has been shown that inhibition of NF-*κ*B is an effective approach to inhibit osteoclast formation and bone resorptive activity [40]. The vascular endothelial growth factor A (*VEGFA*) gene is located on chromosome 6p31.3 [41]. It encodes a member of vascular endothelial growth factor. Several previous studies have linked multiple genetic polymorphisms within the promoter region of *VEGFA* to the disease status of nontraumatic ONFH. The *STAT1* signaling pathway is strongly activated in the pathogenesis and progression of osteoporosis [42]. Acceleration in fracture callus remodeling and membranous ossification has been observed in *STAT1*-deficient mice [43].

The mechanisms of action and molecular targets of the THSWT formula for ONFH were explored using a network pharmacology approach in this study. Kaempferol, luteolin, and baicalein regulated the most number of targets associated with ONFH. The THSWT formula may regulate osteocyte function through specific BPs, including responses to toxic substances and oxidative stress. The regulated pathways include the relaxin, focal adhesion, NF-*κ*B, TLR, and AGE/RAGE signaling pathways. *RELA*, *VEGFA*, and *STAT1* are the important target genes in the gene network of the THSWT formula for the treatment of ONFH.

## Figures and Tables

**Table 1:** The list of active compounds in the THSWT formula.

Chinese name	Latin name	Species	Family	TCMSP ID	Chemical compounds	Oral bioavailability (%)	Drug-likeness
Bai-Shao	*Paeoniae Radix Alba*	*Paeonia lactiflora Pall*	*Ranunculaceae*	MOL000211	Mairin	55.38	0.78
				MOL000358	*β*-sitosterol	36.91	0.75
				MOL001359	Sitosterol	36.91	0.75
				MOL000422	Kaempferol	41.88	0.24
				MOL000492	(+)-catechin	54.83	0.24
				MOL001910	11*α*, 12*α*-epoxy-3*β*-23-dihydroxy-30-norole-an-20-en-28, 12*β*-olide	64.77	0.38
				MOL001918	Paeoniflorgenone	87.59	0.37
				MOL001919	(3*S*, 5*R*, 8*R*, 9*R*, 10*S*, 14*S*) -3, 17-dihydroxy-4, 4, 8, 10, 14-pentamethyl-2, 3, 5, 6, 7, 9-hexahydro-1H-cyclopenta[a]phenanthrene-15, 16-dione	43.56	0.53
				MOL001921	Lactiflorin	49.12	0.80
				MOL001924	Paeoniflorin	53.87	0.79
				MOL001925	paeoniflorin_qt	68.18	0.40
				MOL001928	albiflorin_qt	66.64	0.33
				MOL001930	Benzoyl paeoniflorin	31.27	0.75
Dang-Gui	*Angelicae Sinensis Radix*	*Angelica sinensis (Oliv.) Diels*	*Apiaceae*	MOL000358	*β*-sitosterol	36.91	0.75
				MOL000449	Stigmasterol	43.83	0.76
Tao-Ren	*Persicae Semen*	*Prunus persica (L.) Batsch*	*Rosaceae*	MOL000358	*β*-sitosterol	36.91	0.75
				MOL001323	Sitosterol alpha1	43.28	0.78
				MOL001328	2,3-didehydro GA70	63.29	0.50
				MOL001329	2,3-didehydro GA77	88.08	0.53
				MOL001339	GA119	76.36	0.49
				MOL001340	GA120	84.85	0.45
				MOL001342	GA121-isolactone	72.70	0.54
				MOL001343	GA122	64.79	0.50
				MOL001344	GA122-isolactone	88.11	0.54
				MOL001348	Gibberellin 17	94.64	0.49
				MOL001349	4a-formyl-7alpha-hydroxy-1-methyl-8-methylidene-4aalpha,4bbeta-gibbane-1alpha,10beta-dicarboxylic acid	88.60	0.46
				MOL001350	GA30	61.72	0.54
				MOL001351	Gibberellin A44	101.61	0.54
				MOL001352	GA54	64.21	0.53
				MOL001353	GA60	93.17	0.53
				MOL001355	GA63	65.54	0.54
				MOL001358	Gibberellin 7	73.80	0.50
				MOL001360	GA77	87.89	0.53
				MOL001361	GA87	68.85	0.57
				MOL001368	3-O-p-coumaroylquinic acid	37.63	0.29
				MOL001371	Populoside_qt	108.89	0.20
				MOL000296	Hederagenin	36.91	0.75
				MOL000493	Campesterol	37.58	0.71
Shu-Di-Huang	*Rehmanniae Radix Praeparata*	*Rehmannia glutinosa Libosch*	*Scrophulariaceae*	MOL001359	Sitosterol	36.91	0.75
				MOL000449	Stigmasterol	43.83	0.76
Chuang-Xiong	*Chuanxiong Rhizoma*	*Ligusticum chuanxiong Hort*	*Apiaceae*	MOL001359	Sitosterol	36.91	0.75
				MOL000433	Folic acid	68.96	0.71
				MOL001494	Mandenol	42.00	0.19
				MOL002135	Myricanone	40.60	0.51
				MOL002140	Perlolyrine	65.95	0.27
				MOL002151	Senkyunone	47.66	0.24
				MOL002157	Wallichilide	42.31	0.71
Hong-Hua	*Carthami Flos*	*Carthamus tinctorius L.*	*Compositae*	MOL000358	*β*-sitosterol	36.91	0.75
				MOL000449	Stigmasterol	43.83	0.76
				MOL000422	Kaempferol	41.88	0.24
				MOL001771	Poriferast-5-en-3beta-ol	36.91	0.75
				MOL002694	4-[(E)-4-(3,5-dimethoxy-4-oxo-1-cyclohexa-2,5-dienylidene) but-2-enylidene]-2,6-dimethoxycyclohexa-2,5-dien-1-one	48.47	0.36
				MOL002680	Flavoxanthin	60.41	0.56
				MOL002695	Lignan	43.32	0.65
				MOL002698	Lupeol-palmitate	33.98	0.32
				MOL002706	Phytoene	39.56	0.50
				MOL002707	Phytofluene	43.18	0.50
				MOL002710	Pyrethrin II	48.36	0.35
				MOL002712	6-Hydroxykaempferol	62.13	0.27
				MOL002714	Baicalein	33.52	0.21
				MOL002717	qt_carthamone	51.03	0.20
				MOL002719	6-Hydroxynaringenin	33.23	0.24
				MOL002721	Quercetagetin	45.01	0.31
				MOL002757	7,8-dimethyl-1H-pyrimido[5,6-g]quinoxaline-2,4-dione	45.75	0.19
				MOL002773	Beta-carotene	37.18	0.58
				MOL002776	Baicalin	40.12	0.75
				MOL000006	Luteolin	36.16	0.25
				MOL000953	CLR	37.87	0.68
				MOL000098	Quercetin	46.43	0.28

**Table 2:** The list of targets and active compounds of Tao-Hong-Si-Wu-Tang formula against ONFH.

TCMSP ID	Chemical compound	Herb source	Target
MOL000006	Luteolin	*Carthami Flos*	PTGS2, PRSS1, RELA, VEGFA, CCND1, MMP2, RB1, JUN, IL6, MMP1, PCNA, MCL1, PTGES, MET
MOL000296	Hederagenin	*Persicae Semen*	ADH1C, SCN5A, PTGS2
MOL000358	Gibberellin 7	*Paeoniae Radix Alba*, *Angelicae Sinensis Radix*, *Carthami Flos*, *Persicae Semen*	PTGS2, SCN5A, CHRM4, ADRA1A, OPRM1, JUN, PRKCA
MOL000422	Kaempferol	*Paeoniae Radix Alba*, *Carthami Flos*	PTGS2, PRSS1, ACHE, F7, CALM1, RELA, JUN, MMP1, STAT1, VCAM1, CYP1B1, ALOX5, HAS2, AHR, NR1I3
MOL000449	Stigmasterol	*Rehmanniae Radix Praeparata*, *Angelicae Sinensis Radix*, *Carthami Flos*	ADH1C, PTGS2, SCN5A, ADRA1A
MOL000492	(+)-catechin	*Paeoniae Radix Alba*	PTGS2, CALM1, HAS2
MOL000493	Campesterol	*Persicae Semen*	PTGS2
MOL001323	Sitosterol alpha1	*Persicae Semen*	PTGS2, ADH1C
MOL001328	2,3-didehydro GA70	*Persicae Semen*	PTGS2, PRSS1
MOL001329	2,3-didehydro GA77	*Persicae Semen*	PTGS2
MOL001340	GA120	*Persicae Semen*	PTGS2
MOL001352	GA54	*Persicae Semen*	PTGS2, CALM1
MOL001355	GA63	*Persicae Semen*	PTGS2
MOL001358	Gibberellin 7	*Persicae Semen*	PTGS2
MOL001361	GA87	*Persicae Semen*	PTGS2
MOL001368	3-O-p-coumaroylquinic acid	*Persicae Semen*	PTGS2, CALM1
MOL001494	Mandenol	*Chuanxiong Rhizoma*	PTGS2
MOL001924	Paeoniflorin	*Paeoniae Radix Alba*	IL6, CD14, LBP
MOL002135	Myricanone	*Chuanxiong Rhizoma*	SCN5A, PTGS2, F7
MOL002140	Perlolyrine	*Chuanxiong Rhizoma*	PTGS2
MOL002157	Wallichilide	*Chuanxiong Rhizoma*	PTGS2, NR3C1
MOL002694	4-[(E)-4-(3,5-dimethoxy-4-oxo-1-cyclohexa-2,5-dienylidene) but-2-enylidene]-2,6-dimethoxycyclohexa-2,5-dien-1-one	*Carthami Flos*	PTGS2
MOL002695	Lignan	*Carthami Flos*	PTGS2, CALM1
MOL002710	Pyrethrin II	*Carthami Flos*	PTGS2
MOL002712	6-hydroxykaempferol	*Carthami Flos*	PTGS2, PRSS1
MOL002714	Baicalein	*Carthami Flos*	PTGS2, PRSS1, CALM1, RELA, VEGFA, AHR, EGLN1, APOD
MOL002717	qt_carthamone	*Carthami Flos*	PTGS2
MOL002721	Quercetagetin	*Carthami Flos*	PTGS2, PRSS1
MOL002757	7,8-dimethyl-1H-pyrimido[5,6-g]quinoxaline-2,4-dione	*Carthami Flos*	PTGS2
MOL002773	Beta-carotene	*Carthami Flos*	VEGFA, MMP2, JUN, PTGS2, MMP1, CAV1, GJA1

**Table 3:** Topological parameters of candidate compounds for THSWT against ONFH.

TCMSP ID	Chemical compound	Degree	Betweenness	Closeness
MOL000422	Kaempferol	15	1080.90	36.32
MOL000006	Luteolin	14	1370.81	36.17
MOL002714	Baicalein	8	394.46	31.65
MOL000358	Gibberellin 7	7	503.66	30.98
MOL002773	Beta-carotene	7	365.76	30.98
MOL000449	Stigmasterol	4	120.36	28.98
MOL000296	Hederagenin	3	66.29	28.32
MOL002135	Myricanone	3	65.72	28.32
MOL000492	(+)-catechin	3	49.35	28.32
MOL001924	Paeoniflorin	3	258.00	19.03
MOL002157	Wallichilide	2	130.00	27.65
MOL001323	Sitosterol alpha1	2	38.77	27.65
MOL001352	GA54	2	11.67	27.65
MOL001368	3-O-p-coumaroylquinic acid	2	11.67	27.65
MOL002695	Lignan	2	11.67	27.65
MOL001328	2,3-didehydro GA70	2	9.63	27.65
MOL002712	6-hydroxykaempferol	2	9.63	27.65
MOL002721	Quercetagetin	2	9.63	27.65
MOL001329	2,3-didehydro GA77	1	0.00	26.98
MOL001340	GA120	1	0.00	26.98
MOL001355	GA63	1	0.00	26.98
MOL001358	Gibberellin 7	1	0.00	26.98
MOL001361	GA87	1	0.00	26.98
MOL000493	Campesterol	1	0.00	26.98
MOL001494	Mandenol	1	0.00	26.98
MOL002140	Perlolyrine	1	0.00	26.98
MOL002694	4-[(E)-4-(3,5-dimethoxy-4-oxo-1-cyclohexa-2,5-dienylidene) but-2-enylidene]-2,6-dimethoxycyclohexa-2,5-dien-1-one	1	0.00	26.98
MOL002710	Pyrethrin II	1	0.00	26.98
MOL002717	qt_carthamone	1	0.00	26.98
MOL002757	7,8-dimethyl-1H-pyrimido[5,6-g]quinoxaline-2,4-dione	1	0.00	26.98

## Data Availability

Raw data were generated at Wangjing Hospital. Derived data supporting the findings of this study are available from the corresponding authors on request.
